# Supplementary material for: Development and reliability testing of a qualitative observational rating system for individuals with brachial plexus injury performing functional capacity evaluation tests
Source: PLoS One. 2026 Apr 13;21(4):e0345464. doi: 10.1371/journal.pone.0345464 (PMC13075681; doi:10.1371/journal.pone.0345464)
Supplement: S1 Table — (DOCX) [file pone.0345464.s001.docx]

**S1 Table. Physical examination: assessed range of movement and muscles selected for strength testing.**

| **Joint** | **Assessed movements** | **Selected muscles for testing** |
| --- | --- | --- |
| Shoulder | Abduction  External rotation | Rhomboid muscle  Supraspinatus muscle  Infraspinatus muscle  Serratus anterior  Deltoid muscle |
| Elbow | Flexion  Extension | Biceps  Triceps |
| Wrist | Dorsal flexion  Palmar flexion  Pronation  Supination | Wrist extensors  Wrist flexors |
| Hand | Extension  Flexion  Opposition of the thumb  Intrinsic plus position | Extensor digitorum muscle (2^nd^ digit) Flexor digitorum superficialis muscle (2^nd^  digit).  Opponens pollicis  Abductor digiti minimi  Adductor pollicis muscle |

The sensation of the hand was assessed using The Semmes-Weinstein monofilament filaments. Each individual's thumb, index finger, and little finger were touched three times on the palmar side of the hand. The monofilaments used for testing were 2.83, 3.61 and 4.31.

Results of the physical of the included individuals with BPI (n=15):

Full range of motion of all examined joints (n=4): of which three individuals had a strength of 4 or 5 on the Medical Research Council (MRC) scale of all tested muscles of one individual had a strength of MRC 3 or 4 in all tested muscles. The sensation of the hand was diminished for light touch in all four individuals with a full active range of motion.

Diminished range of motion in at least one joint (n=11): caused by contractures (5 individuals) or a limited strength (MRC < 3, 6 individuals). Two of these individuals had an intact sensation of the hand, eight individuals had a diminished sensation for light touch and one had a diminished protective sensation of the hand.
