# Supplementary material for: Development and reliability testing of a qualitative observational rating system for individuals with brachial plexus injury performing functional capacity evaluation tests
Source: PLoS One. 2026 Apr 13;21(4):e0345464. doi: 10.1371/journal.pone.0345464 (PMC13075681; doi:10.1371/journal.pone.0345464)
Supplement: S2 Table — (DOCX) [file pone.0345464.s002.docx]

**S5 Table. Results of the Qualitative evaluation phase**

| FCE-OH test | Video | Rating session 1:  Number of raters scored compensation (n= 17) | Rating session 2:  Number of raters scored compensation  (n= 16) |
| --- | --- | --- | --- |
| Overhead working test | 1 | 17 | 15 |
|  | 2 | 6 | 0 |
|  | 3 | 17 | 16 |
|  | 4 | 14 | 15 |
|  | 5 | 16 | 16 |
|  | 6 | 1 | 2 |
|  | 7 | 17 | 16 |
|  | 8 | 0 | 0 |
| Average number of videos rated as not within normal limits (max = 8) |  | 5.2 | 4.7 |
| Repetitive reaching test | 1 | 2 | 2 |
|  | 2 | 16 | 16 |
|  | 3 | 8 | 9 |
|  | 4 | 16 | 14 |
|  | 5 | 16 | 16 |
|  | 6 | 8 | 7 |
|  | 7 | 16 | 16 |
|  | 8 | 0 | 0 |
| Average number of videos rated as not within normal limits (max = 8) |  | 4.8 | 5.0 |
| Fingertip dexterity test | 1 | 1 | 1 |
|  | 2 | 3 | 1 |
|  | 3 | 17 | 16 |
|  | 4 | 4 | 7 |
|  | 5 | 3 | 0* |
|  | 6 | 13 | 13* |
|  | 7 | 17 | 16 |
|  | 8 | 3 | 1 |
| Average number of videos rated as not within normal limits (max = 8) |  | 3.6 | 3.4 |
| Overhead lifting test two-handed | 1 | 8 | 8 |
|  | 2 | 5 | 6 |
|  | 3 | 11 | 9 |
|  | 4 | 17 | 16 |
|  | 5 | 4 | 1 |
|  | 6 | 10 | 9 |
|  | 7 | 15 | 14 |
|  | 8 | 5 | 5 |
| Average number of videos rated as not within normal limits (max = 8) |  | 4.4 | 4.2 |
| Overhead lifting test one-handed | 1 | 6 | 4* |
|  | 2 | 16 | 15* |
|  | 3 | 13 | 10* |
|  | 4 | 2 | 0* |
|  | 5 | 2 | 2* |
|  | 6 | 10 | 7* |
|  | 7 | 8 | 4* |
| Average number of videos rated as not within normal limits (max = 7) |  | 3.4 | 2.6 |
| Overall average number over videos rated as not within normal limits (max= 39) |  | 21.4 | 20.3 |

* 1 missing data
