# Supplementary material for: Development and reliability testing of a qualitative observational rating system for individuals with brachial plexus injury performing functional capacity evaluation tests
Source: PLoS One. 2026 Apr 13;21(4):e0345464. doi: 10.1371/journal.pone.0345464 (PMC13075681; doi:10.1371/journal.pone.0345464)
Supplement: S1 Fig — (DOCX) [file pone.0345464.s003.docx]

**S2 Fig.** **Flow chart of the construction phase of the development of the scoring system for rating postures and movement patterns in individuals with BPI during the performance of FCE-one handed tests.**

<Insert S2 fig.tiff>

The scoring system was adjusted based on pilot test results and feedback of the raters

*Abbreviations:* BPI, brachial plexus injury; FCE, functional capacity evaluation; ĸ , Fleiss kappa.
