# Supplementary material for: Development and reliability testing of a qualitative observational rating system for individuals with brachial plexus injury performing functional capacity evaluation tests
Source: PLoS One. 2026 Apr 13;21(4):e0345464. doi: 10.1371/journal.pone.0345464 (PMC13075681; doi:10.1371/journal.pone.0345464)
Supplement: S1 File — (DOCX) [file pone.0345464.s004.docx]

**S3 File. Questionnaire qualitative evaluation phase**

Instruction, documents and videos

1. Could you open all documents attached in the e-mail  Yes No
2. Did you have access to all videotapes in the Video Fragment Rating system  Yes No
3. If you were unable to open the documents and/or videotapes, what problems did you encounter? …..

………………………………………………………………………………………………………………………………………………

1. Were the instruction document and instruction video clear?  Yes No
2. Do you have suggestions for improvement of the instruction document and/or instruction video? ………………………………………………………………………………………………………………………..

……………………………………………………………………………………………………………………………………

Scoring system

1. Were the text and photos used in the scoring system clear?  Yes No
   1. If not, what was unclear?..................................................................................................

………………………………………………………………………………………………………………………………………………………………………………………………………………………………………………………………………………………………………………………………………………………………………………………………………………

1. The scoring system was easy in use  agree  disagree
2. Do you have any suggestions to improve the scoring system?....................................................

……………………………………………………………………………………………………………………………………………………………………………………………………………………………………………………………………………………………………………………………………………………………………………………………………………………………………………………………………………………………………………………………………………………………………………………………….

1. I think the scoring system is easy to use in daily practice.

I totally agree 1 2 3 4 5 I totally disagree

1. Watching the instruction video is sufficient training to use the scoring system in daily practice.

I totally agree 1 2 3 4 5 I totally disagree

1. Do you have other suggestions for improvement/feedback?...............................................................................................
