# Supplementary material for: Development and reliability testing of a qualitative observational rating system for individuals with brachial plexus injury performing functional capacity evaluation tests
Source: PLoS One. 2026 Apr 13;21(4):e0345464. doi: 10.1371/journal.pone.0345464 (PMC13075681; doi:10.1371/journal.pone.0345464)
Supplement: S2 File — (DOCX) [file pone.0345464.s005.docx]

**S4. Qualitative scoring system for rating posture and movements of the shoulders and trunk in individuals with brachial plexus injury during the performance Functional capacity evaluation one-handed (FCE-OH). ***

*Remark 1. The original version contained images with examples of within normal limits and not within normal limits, because of privacy reasons these images were removed.

**
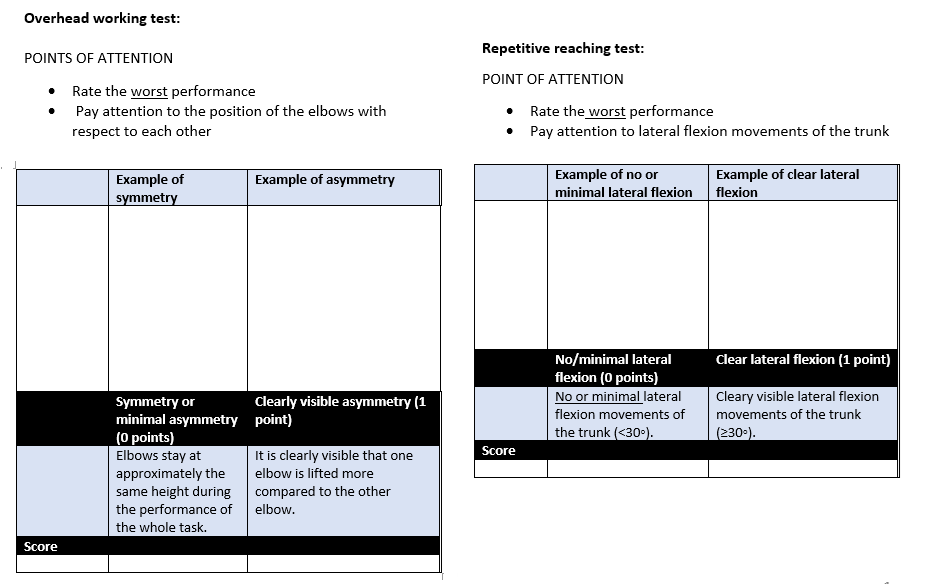
**

**
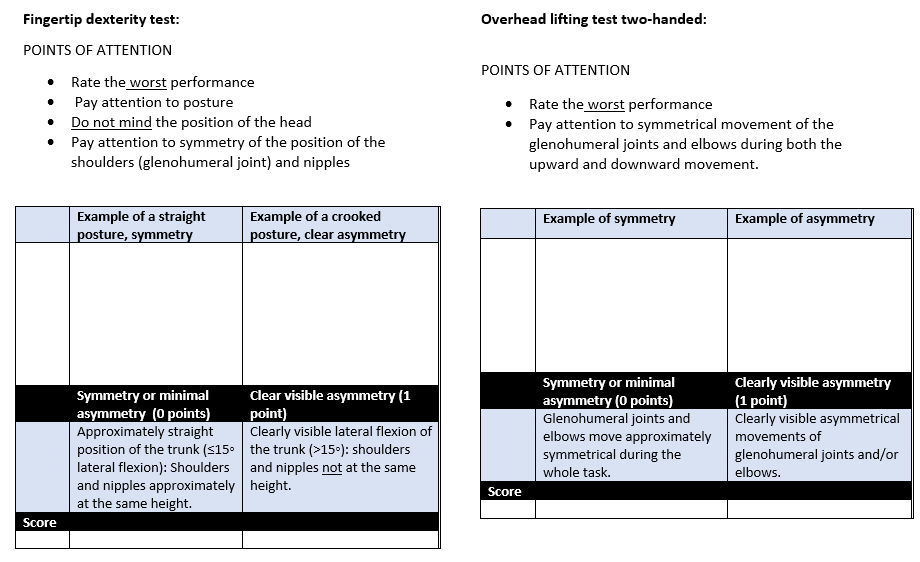
**

**
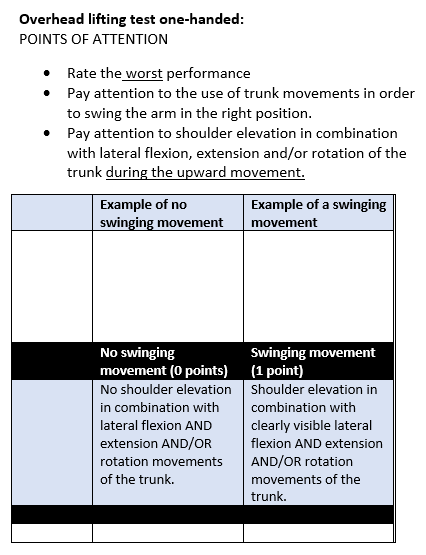
**
