# Supplementary material for: Development and reliability testing of a qualitative observational rating system for individuals with brachial plexus injury performing functional capacity evaluation tests
Source: PLoS One. 2026 Apr 13;21(4):e0345464. doi: 10.1371/journal.pone.0345464 (PMC13075681; doi:10.1371/journal.pone.0345464)
Supplement: Attachment — Submitted filename: Reviewer Report_plos.docx [file pone.0345464.s006.docx]

Reviewer Report

General Assessment and Recommendation

This manuscript addresses an important gap in the evaluation of compensatory movement patterns in individuals with brachial plexus injury (BPI) during one-handed functional capacity evaluation (FCE-OH). The authors propose and test a novel observational scoring system for identifying compensations, and report inter- and intra-rater reliability based on video assessments by 16 raters. The study is original, methodologically sound in many respects, and clinically relevant. However, several methodological, statistical, and reporting issues need clarification and strengthening. I therefore recommend Major Revision.

1. Scope and Novelty

The manuscript is well-positioned: FCE-OH is widely used for work capacity evaluations, yet does not capture how tasks are performed. The observational scoring system adds a qualitative layer that can be highly valuable in preventing musculoskeletal complications due to compensatory movements.

Prior tools developed for prosthesis users are not directly transferable, which justifies the development of a new scale specifically for BPI.

The work is timely and relevant to rehabilitation, occupational health, and clinical ergonomics.

Strengths: International pool of raters, two test sessions to assess stability, item-level analysis, and the addition of an applicability survey.

2. Study Design and Scale Development

Major Revision Request 2.1 – Content validity:

The development process is described but lacks systematic reporting of item generation and expert consensus. How were “within normal limits (WNL)” versus “not WNL” decisions operationalized? The current items are largely symmetry-based; clearer operational definitions and anchor examples (possibly visual diagrams) should be provided in the supplementary material.

Major Revision Request 2.2 – Stratification:

Given the heterogeneity of BPI, stratification by residual function (e.g., ROM, strength categories, lesion levels) is warranted to illustrate item performance across subgroups. This would help clarify whether some items are only reliable in particular clinical subpopulations.

Major Revision Request 2.3 – Training protocol:

Over half of the raters felt that the video-based training was insufficient. A structured training module (e.g., a set of “gold standard” clips with feedback and short quiz) should be outlined, and the improvement from session 1 to session 2 should be explicitly analyzed as a “learning effect.”

3. Statistical Analysis and Reliability

Major Revision Request 3.1 – Kappa limitations:

Low kappa values despite moderate absolute agreement suggest prevalence/bias effects. The authors should include prevalence and bias indices, and/or alternative coefficients such as Gwet’s AC1/AC2 or PABAK.

Major Revision Request 3.2 – Advanced modeling:

If feasible, consider multi-level approaches (e.g., Generalizability Theory or mixed-effects logistic regression), which can simultaneously model variance due to rater, video, and session factors.

Major Revision Request 3.3 – Item-level behavior:

The weakest performance was for overhead lifting items. These may conflate several compensations (shoulder, trunk, elbow). Splitting them into subcomponents could improve clarity and rater agreement.

Revision Request 3.4 – Power and CI width:

Confidence intervals are relatively wide. While the exploratory nature is acknowledged, a brief power calculation or justification of sample size relative to target kappa would add transparency.

4. Video Protocol and Practical Implementation

Several raters reported difficulty due to loss of the sagittal plane in some videos. For future work, multi-angle recordings (front, side, posterior) or camera tracking should be considered.

The instruction that raters could not pause or replay videos mirrors real clinical practice, but should be explicitly justified and included in the user manual.

5. Interpretation of Results

The improvement from session 1 to session 2 suggests a training effect. Quantifying this difference statistically (e.g., confidence intervals for session-to-session change in kappa) would strengthen the claim.

The interpretation of “almost sufficient” reliability should be replaced with numeric ranges and standard thresholds for clarity.

The finding that intra-rater reliability is sufficient while inter-rater reliability is moderate should be highlighted as both a limitation and an opportunity for further rater training.

6. Data Availability and Ethics

Ethics approval and informed consent are clearly stated.

Data availability statement is appropriate. However, I recommend sharing the raw rating matrix, anonymized survey results, rater instructions, and—if ethically permissible—short example video clips in an open repository with DOI. This will greatly facilitate replication and training.

7. Writing, Language, and Presentation

Minor typographical errors should be corrected (e.g., “abled bodied” → “able-bodied,” “prothesis” → “prosthesis”).

Abbreviations should be defined upon first use (e.g., FCE-OH, WNL).

Tables and figures should be polished: abbreviations expanded, statistical measures (κ, CI, % agreement, prevalence) clearly presented.

Supplementary materials should include the final scoring system in a single consolidated PDF.
