# Supplementary material for: Development and reliability testing of a qualitative observational rating system for individuals with brachial plexus injury performing functional capacity evaluation tests
Source: PLoS One. 2026 Apr 13;21(4):e0345464. doi: 10.1371/journal.pone.0345464 (PMC13075681; doi:10.1371/journal.pone.0345464)
Supplement: Attachment — Submitted filename: Response to the reviewers.docx [file pone.0345464.s008.docx]

Dear reviewers,

Thank you for the helpful comments to improve our manuscript. Based on these comments we have modified the text and tables. Below we respond to your comments in detail.

**Reviewer 1**

Major Revision Request 2.1 – Content validity:
The development process is described but lacks systematic reporting of item generation and expert consensus. How were “within normal limits (WNL)” versus “not WNL” decisions operationalized?

- **Response:** Within normal limit was operationalized as: the maximum range of motion deviated maximal two standard deviations from the mean of the control group.

Not within normal limits was operationalized as: the maximum range of motion deviated more than two standard deviations from the mean of the control group or if additional movements were performed that were not observed in the control group, see page 11, line 217-220. The criteria for movements and postures that were classified as “not within normal limits” were based on the criteria we used to develop the ratings system for upper limb prothesis wearers [19]. We added this to the manuscript, page 11, line 220-221. We selected movements and postures that deviated more than 2 standard deviations from the mean range of motion measured in the control group, because with this criterion 95%of the normal movement patterns should be within the selected range. With this criterion we operationally defined movement patterns classified as not within normal limits.

The current items are largely symmetry-based; clearer operational definitions and anchor examples (possibly visual diagrams) should be provided in the supplementary material.

- **Response:** Only the two two-handed FCE-OH tests were assessed based on symmetry; in these FCE-OH tests evident asymmetry was not seen in the control group. For the three one-handed FCE-OH tests clear cutoff points were defined, for example: for the item trunk movements of the repetitive reaching test lateral flexion movements of the trunk >30 degrees were defined as not WNL. In the rating system we used stills of the videos and added lines in order to make clear what was defined as not within normal limits and within normal limits. Although faces of participants were blurred, we cannot attach the rating system because of privacy reasons. Figure 2 shows an example of one item. We added the rating system without the stills of the videos to the supporting information S4).

Major Revision Request 2.2 – Stratification:
Given the heterogeneity of BPI, stratification by residual function (e.g., ROM, strength categories, lesion levels) is warranted to illustrate item performance across subgroups. This would help clarify whether some items are only reliable in particular clinical subpopulations.

- **Response:** Analysis per subgroup was not possible because raters only rated 8 out of the 16 videotapes of individuals with BPI each item, in order to prevent prolonged rating sessions. Although we agree that subgroup analyses might have provided interesting additional information, the subgroups would be too small. Furthermore, we think that analysis of the reliability per subgroup is complicated, because there is not a standard measurement tool that classifies individuals with BPI based on residual function, which complicates the determination of the subgroups. Moreover, it would require different systems for which raters need to train and maintain rating skills for different rating systems (see also discussion page 21 line 419- 424).

Major Revision Request 2.3 – Training protocol:
Over half of the raters felt that the video-based training was insufficient. A structured training module (e.g., a set of “gold standard” clips with feedback and short quiz) should be outlined, and the improvement from session 1 to session 2 should be explicitly analyzed as a “learning effect.”

- **Response:** Thank you for your suggestions, we agree that a structured training module including feedback and practice is needed in further research, this was also suggested in the discussion (page 21, line 408-415).

We suggest that the improvement from session one to session two was caused by a learning effect, but rater specific factors like fatigue, mental health etc. may also have contributed tot the improvement. We did not examine these factors. We added this to the discussion (page 21 line 404-407.

Major Revision Request 3.1 – Kappa limitations:
Low kappa values despite moderate absolute agreement suggest prevalence/bias effects. The authors should include prevalence and bias indices, and/or alternative coefficients such as Gwet’s AC1/AC2 or PABAK.

- **Response:** The interrater reliability kappa values were determined using Fleiss kappa for multiple raters. Determination of prevalence and bias indices are only available for Cohen’s kappa, not for multiple raters kappa (Byrt et al 1993). Also the PABAK statistic is only described for Cohen’s kappa.

We added the alternative Gwet’s AC1/AC2 coefficient which is suitable for multiple raters to Table 3. The results were similar tot Fleiss kappa, which suggests that there were no prevalence effects. We added this to the Result section, page 15, line 338-339.

We also added the average number of videotapes that were rated as “not within normal limits” per FCE-OH test and overall to Table S5 to illustrate the prevalence rates.

Major Revision Request 3.2 – Advanced modeling:
If feasible, consider multi-level approaches (e.g., Generalizability Theory or mixed-effects logistic regression), which can simultaneously model variance due to rater, video, and session factors.

- **Response:** Thank you for your suggestions; however we believe that a multi-level approach is not feasible. We initiated the development of the rating system with the ultimate goal of implementing it in clinical practice, provided that reliability is sufficient. In clinical settings, the rating system must be applied by multiple raters to assess multiple individuals with BPI. Therefore, if variance can be attributed to either the videos or the raters, these factors would not be targeted for intervention.

Major Revision Request 3.3 – Item-level behavior:
The weakest performance was for overhead lifting items. These may conflate several compensations (shoulder, trunk, elbow). Splitting them into subcomponents could improve clarity and rater agreement.

- **Response:** We agree that rating movements of several joints at once may have caused difficulties in rating during these items, we also mentioned this in the discussion (page 20, line 392-397).

In the initials draft of the rating system, joint movements were divided in separate items (see figure S2). However, pilot testing of both the first and second drafts revealed that raters experienced difficulties in evaluating these movements individually which may be caused because movements of several joints were subtle, for example shoulder elevation. Based on our experience developing a rating system for upper limb prosthesis users [19], we learned that subtle deviation movements, such as shoulder elevation, were difficult to asses reliably by observation. In order to rate these more subtle movements, we chose to place greater emphasis on symmetry in the two-handed FCE-OH tests. This adjustment improved the results during pilot testing (page 13, line 293-302).

Revision Request 3.4 – Power and CI width:
Confidence intervals are relatively wide. While the exploratory nature is acknowledged, a brief power calculation or justification of sample size relative to target kappa would add transparency.

- **Response:** we performed a post hoc power analysis. The sample size was sufficient for the overall intrarater reliability and interrater reliability. The sample size was insufficient for the separate analysis of the inter and intra rater reliability of the items.

We added this to the Results section, page 16 line 340-347:The required sample size for the intra-rater reliability analysis (power 0.8, expected kappa 0.6, precision 0.1) was 16 raters, 10 videos. In our study, 16 raters rated 39 videos, which is a sufficient sample size for the overall intra-rater reliability. However, the sample size for individual items was insufficient because only 7 or 8 videos were rated per item by 16 raters, instead of 10 the videos required to achieve sufficient power.

The required sample size for the interrater analysis (power 0.8, expected kappa 0.6, precision 0.1) was 246 ratings. In our study, each video was rated by 16 raters, resulting in 624 ratings, which is sufficient for the overall interrater reliability analysis. However, for the individual items, only 122 (in case 7 videos were rated) or 128 ratings were obtained, instead of the 246 ratings required to achieve sufficient power.

Several raters reported difficulty due to loss of the sagittal plane in some videos. For future work, multi-angle recordings (front, side, posterior) or camera tracking should be considered.
The instruction that raters could not pause or replay videos mirrors real clinical practice, but should be explicitly justified and included in the user manual.

- **Response:** We agree that in future studies multi-angle recordings is desired (see also limitation section page 23, line 458-459).

In the instructions the raters received before each rating session, we included that replay or pausing of the videos was not allowed (page 13 line 265-266).

The improvement from session 1 to session 2 suggests a training effect. Quantifying this difference statistically (e.g., confidence intervals for session-to-session change in kappa) would strengthen the claim.

- **Response**: The width of the confidence intervals for both rating sessions was similar (Table 3), furthermore the confidence intervals of both sessions do overlap. We adjusted text hereabout in the discussion, because there may be a learning effect, but also other unexamined rater specific factors, such as fatigue, mental health, time taken for rating, may have contributed to the improvements between sessions. Page 21 line 405-407.

The interpretation of “almost sufficient” reliability should be replaced with numeric ranges and standard thresholds for clarity.

- **Response**: We agree that the term “almost sufficient” is not clear. The kappa value of the interrater reliability was moderate for both sessions based on the thresholds we used. We adjusted the abstract and removed the term “almost sufficient”, page 3 line 47-48. In the conclusion we added the threshold for kappa, page 26 line 531-533.

The finding that intra-rater reliability is sufficient while inter-rater reliability is moderate should be highlighted as both a limitation and an opportunity for further rater training.

- **Response:** Thank you, we added to the discussion session that the interrater reliability may improve with a more extensive training that incorporates structured feedback. Page 21, line 408-409.

6. Data Availability and Ethics
Ethics approval and informed consent are clearly stated.
Data availability statement is appropriate. However, I recommend sharing the raw rating matrix, anonymized survey results, rater instructions, and—if ethically permissible—short example video clips in an open repository with DOI. This will greatly facilitate replication and training.

- **Response:** Thank you, we will share the raw data and rater instruction after acceptance of the manuscript in an open repository <https://doi.org/10.17605/OSF.IO/8JBPY>. We will not share the videos because of privacy reasons.

7. Writing, Language, and Presentation
Minor typographical errors should be corrected (e.g., “abled bodied” → “able-bodied,” “prothesis” → “prosthesis”).
Abbreviations should be defined upon first use (e.g., FCE-OH, WNL).
Tables and figures should be polished: abbreviations expanded, statistical measures (κ, CI, % agreement, prevalence) clearly presented.
Supplementary materials should include the final scoring system in a single consolidated PDF.

- **Response**: Thank you, we adjusted the typing errors. The abbreviations were defined upon the first use. The Tables and Figures were checked, abbreviations were added where necessary. We added the final rating system without illustrative stills of the videos to supporting information S4 (see also our response to comment 2.1).

**Reviewer 2**

Reviewer #2: 1. Claims & Framing
1.1 Abstract results, discussion and conclusions overstate κ values as “sufficient,” whereas they are best described as moderate (κ≈0.59–0.64).

- **Response**: We agree the interrater reliability was moderate, therefore we revised “almost sufficient” to moderate, see also our response to comment 3.4 of reviewer 1. Based on the cut of points for kappa we used (poor if ĸ ≤ 0.4, moderate if 0.41< ĸ< 0.59, sufficient if 0.6 ≤ ĸ < 0.79 and good if ĸ ≥0.80) the intrarater reliability was sufficient. The cut off points were based on J. Sim et al. 2005, Page 13 line 281-282.

1.2 The novelty of the tool compared with prior prosthesis-user and observational/postural tools is not sharply positioned in discussion and introduction.

- **Response**: Previous studies showed that compensatory movements are task specific (page 5 line 100-102) therefore a tool specifically developed for the tasks of the FCE-OH test was needed.

The aim of the developed rating system for individuals with BPI was the same as for upper limb prosthesis users: to assess shoulder and trunk movements during the performance of FCE-OH tests in a standardized and reliable manner. However the rating system for upper limb prosthesis users appeared not to be applicable to individuals with BPI and was therefore adjusted.

Movement patterns of individuals with BPI that were classified as not within normal limits could not be differentiated from normal movement patterns using the previously developed rating system for upper limb prosthesis users.

In the introduction we added that to the best of our knowledge an observational rating system rating movement patterns applicable for the use in individuals with BPI does not exist, in order to position the novelty of the rating system better (page 6 line 109-110).

In the discussion we added that a new rating system was developed and clarified that the rating system designed for prosthesis wearers was not applicable to individuals with BPI. Page 19 line 374 and page 19 line 380-382.

2. Scale Development (Methods)
2.1 The reliance on one student, one professional, and internal group consensus limits the robustness of the item selection; broader expert involvement (e.g., multiple clinicians or a Delphi panel) would improve validity.

- **Response:** The internal consensus group consisted of 3 rehabilitation physicians and one FCE-expert (this was added to the method section page 11, 228-230). Additionally, raters added suggestions for improvement of items or extra items in the feedback during the construction phase. These suggestions were used to improve the items of rating system. We agree that a Delphi study may result in a rating system with enhanced validity. We added this to the discussion, page 23 line 470-472.

2.2 The choice of a >2 SD threshold is statistical but not clearly justified clinically. Why is this cutoff appropriate for defining abnormal compensation?

- **Response**: We agree that the threshold was as statistical choice, see also our response to comment 2.1 of reviewer 1. Determining a clinically relevant threshold is challenging, as to our knowledge only three previous studies have examined movement patterns in individuals with BPI [2,4]. These studies demonstrated that movement patterns were task specific. None of these studies analyzed movement patterns during FCE-OH tasks or comparable activities.

The chosen threshold may have resulted in that only larger movements were classified as not within normal limits. Clinically we think these movements are the most important to intervene, because these movements cause larger internal moments around joints and require therefore more muscle force. These movements may therefore cause muscle fatigue and thereby increase the risk of developing musculoskeletal complaints [5]. We added this to the limitation section of the discussion, page 22 and 23 line 448-455.

2.3 The binary WNL/nWNL scoring system is simple and practical, but may oversimplify nuanced compensatory behaviors. A graded scale (e.g., mild/moderate/severe deviation) could capture variation more effectively.

- **Response**: We agree that the rating system is not suitable for capturing nuances in compensatory behaviors. During the development of the observational rating system for upper limb prosthesis wearers, the initial drafts included graded scales with the categories “no compensation”, “subtle compensation” and “strong compensation “[19]. However, because the raters experienced difficulties distinguishing between the categories “no compensation” and “subtle compensation” (Page12 line 238-242), we decided to develop a dichotomous rating system. We believe that identifying the more severe deviating movements is the most important, because these movements are most relevant for intervention. Individuals with a limited function in one upper limb will always need to compensate, but the extent to which these compensatory strategies are used varies between individuals and can be optimized.

3. Raters, Training, and Blinding (Methods)
3.1 The manuscript does not describe how raters were trained, calibrated, or whether their backgrounds (clinical vs non-clinical) might have influenced ratings. This is a crucial omission, as interrater reliability depends heavily on training consistency.

- **Response**: We agree that training and backgrounds of the raters are essential for interrater reliability. Details regarding the raters’ clinical backgrounds, duration of work experience and FCE-experience were already included in the results section of the manuscript (page 14 and 15 line 309-316). Before the rating sessions all raters received identical training, consisting of the video instruction (page 12 line 257 -258).

To address the concern regarding potential influence of prior FCE experience, we performed an analysis comparing interrater reliability between raters with and without FCE experience (Page 15, line 330-333 and line 335-337). The results were similar:
• First rating session: raters with FCE experience κ = 0.56 (95% CI 0.41–0.70), raters without FCE experience κ = 0.53 (95% CI 0.39–0.66).
• Second rating session: raters with FCE experience κ = 0.64 (95% CI 0.56–0.73), raters without FCE experience κ = 0.58 (95% CI 0.43–0.73).

These findings suggest that prior FCE experience did not substantially influence interrater reliability.

Because all raters had a clinical background with expertise in musculoskeletal assessment and received the same instructions prior rating, we did expect differences in rating patterns and therefore we did not perform subgroup analyses between different professional groups.

3.2 It is unclear whether raters were blinded to diagnosis (BPI vs control). Knowledge of group assignment could bias assessments.

- **Response**: We have added to the method section that only videos of individuals with BPI were rated (page 12 line 248-250). All raters were informed that the videos they rated depicted individuals with BPI.

3.4 The questionnaire is only briefly mentioned. No details are provided regarding its design, validation, or analysis. Without transparency, these findings cannot be meaningfully interpreted.

- **Response**: The questionnaire we used in the qualitative evaluation phase was a self-developed questionnaire. This questionnaire had previously been used during the development of the rating system for upper limb prosthesis wearers, but has not been formally validated. Only descriptive analyses were performed on the data obtained from this questionnaire. We added this to the method section page 13 line 268-272 and line 286-287.

4. Sample Size & Power (Methods)
4.1 The sample size (15 BPI, 21 controls, 16 raters) is modest. No power calculation or justification is provided. Reliability studies require such justification to ensure κ estimates are stable.

- **Response**: A power analysis to determine the minimal number of individuals with BPI needed for the development of the rating system was not possible because the variance of movement patterns and postures of individuals with BPI was unknown. Post hoc we checked if saturation of the observed movement patterns and postures was reached for each FCE-OH test (Page 23, line 473-477). Saturation was reached if no new deviating movement patterns and postures were observed in at least the last four analyzed videotapes. In four out of five FCE-OH tests saturation of the observed deviating movement patterns and postures was reached. Only for the overhead lifting test, no saturation was reached. For the controls saturation was reached for all FCE-OH tests.

Furthermore, we performed a post hoc power analysis which indicated that the sample size was sufficient for the overall intrarater reliability and interrater reliability, see also our response to comment 3.4 of reviewer 1. We added this to the Results section, page 16 line 340-347.

5. Statistics & Interpretation
5.1 The Results section is statistically adequate but lacks depth: (1. Were reliability values consistent across different FCE tasks, or did some tasks show higher agreement? 2. Were there systematic differences in rating patterns between raters with different professional backgrounds? 3. Were there differences in reliability between the BPI and control groups?)

- **Response**: 1. Table 3 shows that the interrater agreement was lower for both overhead lifting tests compared to the other FCE-OH tests. In contrast the intrarater agreement was only lower for the overhead lifting test one-handed. This was also reported in the Results section on page 15 line 328 and 334-335.

2. There were no differences observed in the rating patterns between raters, we analysed if years of FCE-experience influenced the ratings but this was not the case (see also our response to comment 3.1 of reviewer 2).

3. We did not study this, because this was not the aim of our study. Raters only rated videos of individuals with BPI, we added this to the Methods section, page 12 line 248-250. We only used the videos of controls to determine movements and postures that were within normal limits during the planning phase. In the next phases of scale development the videos of controls were not used.

5.2 The discussion should address why interrater reliability improved from session 1 to session 2. Was this due to learning effects, improved familiarity with the tool, or random variation? Without exploring this, the findings are difficult to contextualize.

- **Response**: We did not analyze the reasons for the improvement in interrater reliability. This may reflect a learning effect, but other unexamined rater-specific factors—such as fatigue, mental health, or time taken for rating—could also have contributed. Alternatively, the change may be due to random variation, as the confidence intervals showed overlap. We added this to the discussion, page 21, line 405-407.

5.3 Although most raters described the tool as “easy to use,” only ~50% considered it clinically feasible, citing insufficient training. This contradiction requires deeper analysis.

- **Response**: Unfortunately we did not ask the raters why they did not agree with the statement that the system would be easy to use in clinical practice. We added this to the discussion, page 22 line 432-434. Insufficient training may be one reason, because only 40% agreed with the statement that watching the video instruction was a sufficient training for the use of the rating system. Another reason may be the difficulties raters experienced during rating which may be caused by variability in movement patterns. See the supporting information (S3) for the questionnaire we used.

6. Language & Structure
6.1 Minor grammar/typography:
The keywords in the PLOS ONE submission system contain a typo and should be carefully checked.
Line 238: “video’s” should be corrected to “videos.”

- **Response**: Thank you, we corrected this. Page 13 line 264

6.2 Consistently use prosthesis (not prothesis).

- **Response**: Thank you, we also corrected this in the manuscript.

6.3 Perform a thorough proofread to correct similar small grammar, pluralization, and capitalization issues throughout the text.

- **Response**: we performed a thorough proofread and made corrections where needed.

6.4 Limitations section:
The Limitations are presented in great detail, but the section currently reads as overly long, repetitive, and weighted with procedural details. While transparency is commendable, the narrative would benefit from focusing on the most impactful issues and reframing them in a forward-looking way.

- **Response**: Thank you, we rewrote the limitation section, page 22-24, line 447-485.

**Reviewer 3**:

1. Can you expand more on the background part on the abstract

- **Response**: we added to the background part of the abstract that “Quantifying compensatory strategies of these individuals may help to minimize the use of dysfunctional compensatory strategies and prevent musculoskeletal complaints “, page 2 line 23-25

2. In the introduction part, "Individuals with brachial plexus injury (BPI) need to compensate for the loss of function of the affected arm." I suggest first to give a brief mechanism of action and intervention of the brachial plexus anatomy and physiology

- **Response**: Thank you for your suggestion, we added a brief explanation of BPI to the introduction. Page 4, line 57-63.

3. Can you mention if the able bodied controls aware of the research and that they are being controls or if it was blinded to them?

- **Response**: yes, the able-bodied controls knew they were controls. We added this to the Methods section, page 9 line 192-193.

4. In the mentioned raters part "Twenty raters were recruited using an international Linked-In FCE research network and by contacting medical professionals at our local medical center" can you mention more about the expertise of the raters whether they are MDs or experts in Orthopedics or Physical Therapy.

- **Response**: this was reported in the results section (page 14 and 15, line 309-316). Eight raters were physiotherapists, five rehabilitation physicians, two occupational therapists and two residents for rehabilitation medicine.

5. From this part "Recruitment of the raters took place from 19 November 2021 to 1 February 2022" can you mention also the time frame of observing movement patterns

- **Response**: the rating sessions were conducted between 17 January 2022 till 2 March 2022, we added this to the Results section, page 15 line 321-322.

6. In Line 148 "Video recordings of 15 individuals with BPI performing FCE-OH tests (2 females, 13 males, mean age 49.9 ± 10.9 years, mean time since onset BPI 9.6 ± 9.7 years, recorded during a previous study that aimed to determine the functional capacity of individuals with BPI" i suggest and encourage the authors to provide a table for better visualization and show data.

- **Response**: Thank you for your suggestion, we have presented the data in Table 1 for better visualization.

7. Also in Line 153-154 "four had a full active range of motion of the affected limb and eleven had a diminished active range of motion of at least one joint caused by contractures or limited strength. also a table here will lead to a better visualization.

- **Response**: We have also presented this data in Table 1.

8. Same Applies to line 169

- **Response**: We have also presented this data in Table 1.

9. Please reorganize wording and clarity in here "Only three out of eight items of the rating system for upper limb prosthesis wearers were suitable for use in individuals with BPI (overhead lifting test two211 handed; items trunk and shoulder movements and fingertip dexterity test; item trunk movements)" as which were the 3 items used.

- **Response**: We reorganized the wording to “ Only three out of eight items of the rating system for upper limb prosthesis wearers were suitable for use in individuals with BPI. These items included (1) trunk and (2) shoulder movements from the two-handed overhead lifting test, as well as (3) trunk movements from the fingertip dexterity test.” Page 11 line 230-233.

10. in Line 223 " Some video recordings appeared to be more difficult to rate than others. In order to reduce the influence of one single video recording on the results, eight video fragments were rated in pilot test 3, 4 and 5. Raters of these pilot tests were also asked to rate the certainty of their ratings (dichotomous, certain: yes or no), in order to identify difficulties in rating." Were single Videos was evaluated by one rater only? if yes can you mention that in the limitations section as operator bias might present.

- **Response**: No, all the selected video fragments were evaluated by all the raters in the pilot tests and in the qualitative evaluation phase. We added to the manuscript that all raters participating in the pilot tests rated 8 video fragments per FCE-OH test. Page 12, line 247-250.

11. In Line 318, Feasibility and usability I would also encourage having a diagram, table, or chart presenting the data.

- **Response**: Thank you, we have added Table 4 to the manuscript which presents the data. Page 19.

12**.** Great Use of the matrix of discussion. a one point to add is just comparing current study findings to the two studies that evaluated the use of compensatory movements you mentioned before

- **Response**: Thank you. We added to the discussion that the variability in movement patterns we observed was also observed by Mahon et al. [4] (Page 20 line 387-388). We could not compare the observed deviating movements and postures, because the previous studies showed that movement patterns are task specific [2,4]. However, in the limitation section we noted that not all the smaller deviating movements (like scapulothoracic movements, rotation of the humerus and movement of the scapula and clavicle with respect to the thorax) can be detected using the developed observational rating system. These movements were observed in the previous two studies, which used motion capture systems. Page 23 line 465-468.

13. I suggest restructuring the discussion and limitation parts as limitation part have an enormous amount of paragraphs suitable in the discussion

- **Response**: we agree and we rewrote the limitation section, page 22-24, line 447-485.
